# Concomitant Fahr’s syndrome and thoracic ossification of the posterior longitudinal ligament caused by idiopathic hypoparathyroidism – case report

**DOI:** 10.1186/s12891-019-2747-1

**Published:** 2019-08-07

**Authors:** Ikchan Jeon, Kyu Hyang Cho, Sang Woo Kim

**Affiliations:** 1 Department of Neurosurgery, Yeungnam University College of Medicine, 170, Hyeonchung street, Nam-Gu, Daegu, 42415 South Korea; 2 Department of Nephrology, Yeungnam University Hospital, Daegu, South Korea

**Keywords:** Fahr’s syndrome, OPLL, Hypoparathyroidism, Hypocalcemia

## Abstract

**Background:**

Fahr’s syndrome presenting multiple and symmetric calcification of basal ganglia and cerebral cortex is rare, and idiopathic hypoparatyroidism is known as one of the causes. The relationship between ossification of posterior longitudinal ligament (OPLL) and idiopathic hypoparatyroidism is also reported in a few cases. Here, we report a patient presenting concomitant Fahr’s syndrome and thoracic OPLL developed by idiopathic hypoparatyroidism.

**Case presentation:**

53-year-old female patient presented myelopathic sign including gait disturbance and both leg weakness (Grade 3) for 4 months after slip down, and has the history of anti-epileptic medication for several years. Magnetic resonance imaging revealed cord compression by the mixed-type OPLL from T5 to T9, and decompressive surgery was planned. Sudden onset generalized tonic-clonic seizure attack developed before the surgery. Hypocalcemia (3.7 mg/dL) with QT prolongation on electrocardiogram, hypomagnesemia (1.4 mg/dL), hyperphosphatemia (7.7 mg/dL), hypoparathyroidism, and normal range of vitamin D was noted. Brain study showed Fahr’s syndrome with multiple and symmetric calcification of basal ganglia, cerebral cortex, and cerebellum. Decompressive laminectomy was performed after transient correction of hypocalcemia. The myelopathic symptoms improved to normal walking by the 14-month follow-up. The cause of hypoparathyroidism was concluded to be idiopathic.

**Conclusion:**

Concomitant expression of Fahr’s syndrome and OPLL related with idiopathic hypoparatyroidism is very rare. However, we recommend considering the possibility of hypoparathyroidism and Fahr’s syndrome when we evaluate the patients with OPLL to avoid the risks of sudden onset seizure and cardiac arrhythmia due to cerebral lesions and hypocalcemia.

## Background

Fahr’s syndrome accompanied by multiple symmetric intracranial calcifications, such as those of the basal ganglia, cerebral cortex, and cerebellum, is rare with a prevalence of < 1/1000,000. Idiopathic hypoparathyroidism is known to be the most common cause among several etiologies including endocrine disorders, adult-onset neurodegenerative conditions, congenital neurological disorders, and inherited or early onset syndrome [1].

Ossification of the posterior longitudinal ligament (OPLL) is one type that includes calcification of the paravertebral ligaments; this has a higher incidence in Asians with the prevalence of 0.4 to 3.0% than other ethnic groups [2]. The relationship between the formation of OPLL and quantitative changes in parathyroid hormone remain unclear. However, there are many reports of the ossification of paravertebral ligaments associated with hypoparathyroidism [3, 4].

Calcification of the brain and intraspinal canal ligament can lead to the development of neurological symptoms such as seizure with Fahr’s syndrome and myelopathy with OPLL. Here, we report a patient presenting with seizure and paraplegia with concomitant Fahr’s syndrome and thoracic OPLL caused by idiopathic hypoparathyroidism.

### Case presentation

**Fig. 1:**
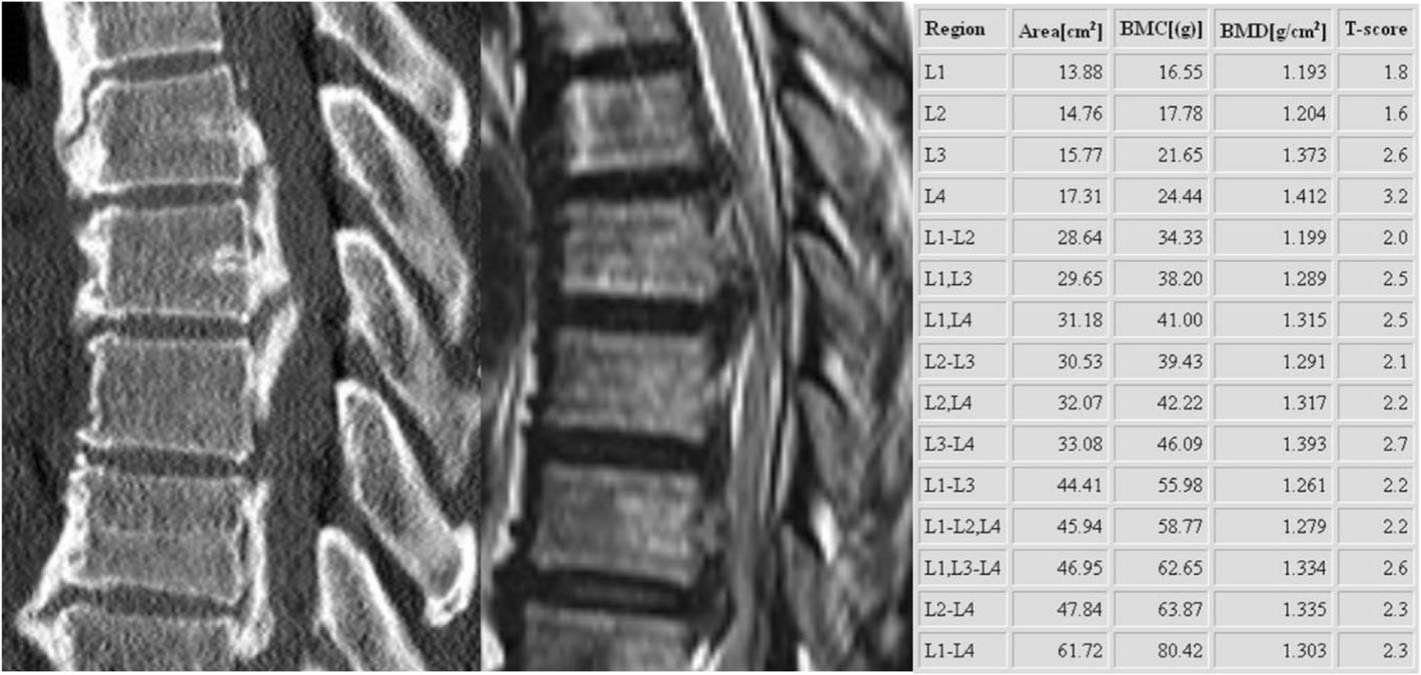
A 53-year-old woman presented with a 4-month history of gait disturbance and bilateral leg weakness (Grade 3) after falling. Computed tomography scan shows ossification of the posterior longitudinal ligament (OPLL) at T5–9. Thoracic cord compression with signal change is observed on magnetic resonance imaging. Bone mineral density with T-scores is greater than that of the same-aged healthy controls

**Fig. 2:**
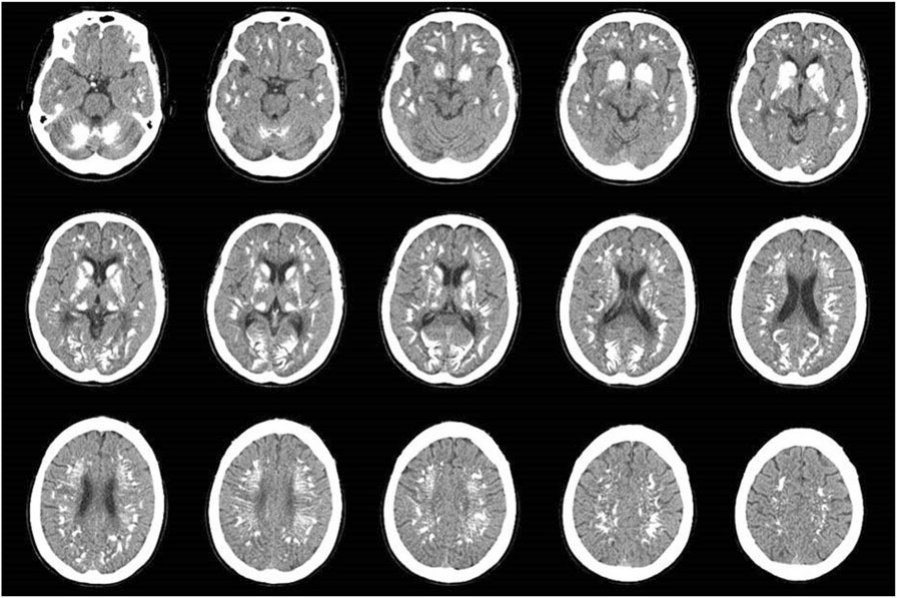
Brain computed tomography scan after generalized tonic-clonic seizure shows multiple symmetric calcifications of the basal ganglia, cerebral cortex, and cerebellum

A 53-year-old woman presented with a four-month history of gait disturbance and bilateral leg weakness (Grade 3) after falling and a history of taking anti-epileptic medication (Valproate sodium 1000 mg/day) for several years. We found increased deep tendon reflexes in the lower extremities on physical examination and signs of thoracic myelopathy in a neurophysiological study. Magnetic resonance imaging and computed tomography revealed thoracic cord compression due to mixed-type OPLL at T5–9 (Fig. 1). Segmental-type OPLL was also observed at C4 without neural compression. Decompressive surgery for the lesion at T5–9 was planned. A sudden onset generalized tonic-clonic seizure developed before the scheduled surgery. Hypocalcemia (3.7 mg/dL, normal range = 8.6–10.6 mg/dL) with QT prolongation on electrocardiogram recording, hypomagnesemia (1.4 mg/dL, normal range = 1.9–3.1 mg/dL), hyperphosphatemia (7.7 mg/dL, normal range = 2.5–4.5 mg/dL), hypoparathyroidism (intact PTH: 2.18 pg/mL, normal range = 15–65 pg/mL), and a normal vitamin D level were found. Brain computed tomography scan showed Fahr’s syndrome with multiple symmetric calcifications of the basal ganglia, cerebral cortex, and cerebellum (Fig. 2). The planned decompressive surgery was performed after achieving temporary normalization following intensive intravenous correction of the hypocalcemia and hypomagnesemia.

The symptoms, which included leg weakness and gait disturbance, improved to normal walking at the 14-month follow-up. During the follow-up period, an endocrinologist and nephrologist at our medical center tried to elucidate the causes of hypoparathyroidism and hypomagnesemia. Hypomagnesemia was normalized with oral magnesium supplementation; and nutritional deficiency was identified as the cause. Unfortunately, neither the cause of the hypoparathyroidism nor sufficient correction of hypocalcemia was achieved, which led to a diagnosis of idiopathic hypoparathyroidism.

## Discussion and conclusion

Hypoparathyroidism is one of the primary causes of hypocalcemia, and can be developed as a result of autoimmune disease, drug use, radiation therapy, tumors, iatrogenic (e.g., surgery-related) reaseon, and hypomagnesemia [5]. Chronic hypocalcemia can lead to ectopic calcification of the central nervous system, such as in Fahr’s syndrome, and its incidence correlates with the duration of untreated hypoparathyroidism [1, 3]. There is no definite therapy that can limit the progression of calcification in Fahr’s syndrome. The existing management strategies focus on symptomatic relief of the clinical manifestations [1, 6, 7]. Medical treatment is needed for neurological disorders including seizure, movement disorders, and psychiatric problems, to control symptoms and prevent deterioration of the patient’s condition. Parathyroid disorders can be resolved by correcting the levels of vitamin D3 and electrolytes, such as calcium, magnesium, and phosphate.

**Fig. 3:**
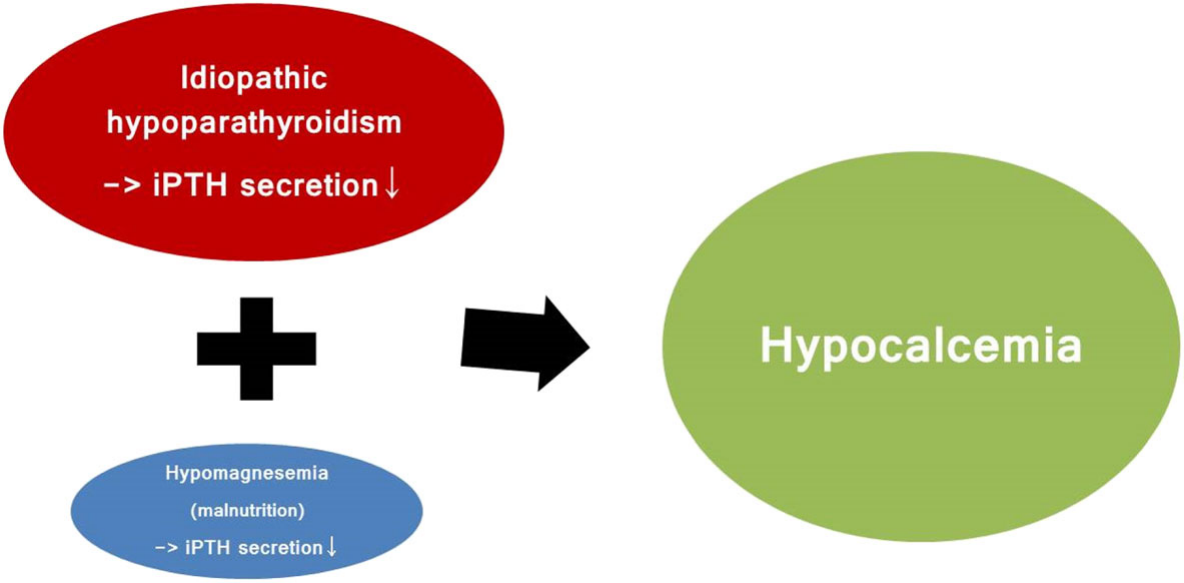
In this case, the primary cause of hypocalcemia was suspected to be idiopathic hypoparathyroidism, with hypomagnesemia due to malnutrition as a secondary correctable cause

Among the causes of hypocalcemia with hypoparathyroidism, hypomagnesemia is usually associated with inadequate oral intake and gastrointestinal or renal loss, which can affect the parathyroid gland, leading to the development of low intact parathyroid hormone (iPTH) and hypocalcemia. Cases of hypocalcemia related to hypomagnesemia are often accompanied by hypokalemia. It is possible to restore both low iPTH and hypocalcemia through magnesium supplementation [7, 8]. Unfortunately, our patient exhibited hypomagnesemia without hypokalemia and no correction of low iPTH and hypocalcemia could be achieved, even with the normalization of hypomagnesemia via oral replacement (Fig. 3).

Unlike in Fahr’s syndrome, the relationship between the formation of paravertebral ligament calcification and parathyroid hormone remains controversial. The incidence of hypoparathyroidism is not significantly higher among the patients with paravertebral ligament calcification than among normal controls [3]. However, there are several reports of paravertebral ligament calcification related to hypoparathyroidism [3, 9, 10]. The literature has described ligamentum flavum ossification [4, 11] and diffuse idiopathic skeletal hyperostosis (DISH) characterized by ossification of the anterior longitudinal ligament of the spine [12–14] in patients with idiopathic hypoparathyroidism. Unfortunately, the relationship between OPLL and parathyroid hormone is relatively unclear and debatable compared to the other types of paravertebral ligament calcification. Okazaki et al. [3] reported a higher incidence of hypoparathyroidism in OPLL. On the other hand, Kashii et al. [15] showed a relationship between a high levels of iPTH and OPLL. Overall, the effect of hypoparathyroidism in the genesis of paraspinal ligament calcification is persuasive, and the role of hypoparathyroidism is regarded as an aggravating factor rather than a causative one [3].

Concomitant Fahr’s syndrome and OPLL related to idiopathic hypoparathyroidism is incredibly rare. In clinical practice, we are more likely to encounter a patient presented with a type of OPLL than one with Fahr’s syndrome for the first time. There is also no treatment method to stop the progression of OPLL as in Fahr’s syndrome. Patients with cervical or thoracic OPLL typically present with myelopathy and require surgical decompression. We need to consider the possibility of undetected hypoparathyroidism and Fahr’s syndrome when there is an electrolyte imbalance including prominent hypocalcemia. These patients require an electrocardiogram and brain work-up. QT prolongation and Fahr’s syndrome in hypocalcemic condition place patients at risks of uncontrolled arrhythmia and seizures, respectively. Optimal correction of hypocalcemia and protection against seizures with anti-epileptic medication should be done when planning an operation.

## Data Availability

Data sharing is not applicable to this article as no datasets were generated or analyzed during the current study.

## Abbreviations

OPLL: Ossification of the posterior longitudinal ligament
PTH: Parathyroid hormone
